# *Trypanosoma cruzi* infection enhances olfactory response in *Triatoma pallidipennis* Stål (Hemiptera: Triatominae) to compounds potentially useful for insect control

**DOI:** 10.1111/mve.12770

**Published:** 2024-10-14

**Authors:** Berenice Jiménez‐Santiago, Any Laura Flores‐Villegas, Samuel Cruz‐Esteban, Margarita Cabrera‐Bravo, Conchita Toriello

**Affiliations:** ^1^ Departamento de Microbiología y Parasitología, Facultad de Medicina Universidad Nacional Autónoma de México Mexico City Mexico; ^2^ Posgrado en Ciencias Biológicas Universidad Nacional Autónoma de México, Circuito de Posgrados, Ciudad Universitaria Mexico City Mexico; ^3^ Instituto de Ecología, A.C. Centro Regional del Bajío, Red de Diversidad Biológica del Occidente Mexicano. Pátzcuaro, Michoacán Mexico; ^4^ CONAHCYT Ciudad de México Mexico

**Keywords:** chemical attraction, chemical ecology, *Triatoma pallidipennis*, *Trypanosoma cruzi*

## Abstract

In Mexico, *Triatoma pallidipennis* is a major vector of *Trypanosoma cruzi*, the causative agent of Chagas disease. Current efforts are focused on developing attractants to control these vectors, using volatile substances derived from vertebrate hosts or compounds known to attract hematophagous insects. However, the efficacy of these compounds in attracting parasite‐infected triatomines remains to be evaluated. In this study, we assessed the attractant activity of octenol (1‐octen‐3‐ol), nonanal and a mixture of odorants consisting of ammonium hydroxide, lactic acid and hexanoic acid (in a ratio of 1:0.2:0.4 respectively), at concentrations of 1, 10 and 100 ng on the N3, N4 and N5 nymphal stages of *T. pallidipennis*, both infected and non‐infected with *T. cruzi*. We also evaluated the synergistic effect of the most effective compounds and doses. All experiments were performed in a laboratory using a Y‐type glass olfactometer. We found that both infected and non‐infected N3 and N4 nymphs were attracted to low doses of octenol, nonanal and the odorant mixture. Particularly noteworthy was the synergistic effect observed between the odorant mixture and nonanal, which significantly increased attraction of *T. cruzi*–infected individuals. These findings contribute to the development of baited traps utilising these compounds for monitoring triatomines in epidemiological studies or for mass trapping to control these vectors.

## Graphical abstract

- *Triatoma pallidipennis* is one of the main vectors of *Trypanosoma cruzi*, the causative agent of Chagas disease.
- Attractant activity of octenol, nonanal and a mixture of lactic acid, hexanoic acid and ammonium hydroxide on *T. cruzi*–infected and non‐infected *T. pallidipennis* nymphal stages.
- A significant finding of our study was the demonstration of a synergistic effect between the mixture and nonanal, which produced an increased attraction in *T. cruzi*–infected insects.

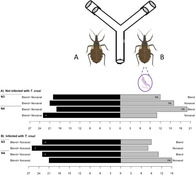

## INTRODUCTION

Chagas disease, a major parasitic disease in Latin America, affecting six to seven million people (WHO, 2023), is caused by *Trypanosoma cruzi* (Chagas) (Kinetoplastida: Trypanosomatidae), a parasitic protozoan transmitted by hematophagous insects of the family Reduviidae, subfamily Triatominae (de Fuentes‐Vicente et al., 2018). Infected triatomines feed on vertebrate hosts and defecate near the bite site, causing pruritus and allowing the host to self‐inoculate by scratching. *T. cruzi* infection can result in cardiac, gastrointestinal and/or neurological disorders (Lidani et al., 2019).

More than 32 species of triatomines have been identified in Mexico, 19 of which are susceptible to infection by *T. cruzi* and are found near to human settlements. *Triatoma barberi* (Usinger), *T. dimidiata* (Latreille) and *T. pallidipennis* (Ståhl) (Hemiptera: Reduviidae) are considered the most important vectors of Chagas disease (Jiménez‐Cortés et al., 2021; Salazar Schettino et al., 2010). *T. pallidipennis* is widely distributed across 13 states in the southeastern, southwestern and central regions of Mexico, and it is commonly found in peri‐urban areas (Jiménez‐Cortés et al., 2021; Martínez‐Ibarra et al., 2012; Salazar‐Schettino et al., 2010). After hatching, triatomines go through five nymphal stages before reaching adulthood. These insects can survive for approximately 250 days when infected with *T. cruzi* in the laboratory (Tay et al., 2008).

Throughout their life cycle, triatomines primarily feed on the blood of various mammals. These insects typically feed at night, using multiple sensory systems to locate potential food sources (Guerenstein & Lazzari, 2009). Since attraction to hosts depends on the detection of host‐emitted chemical signals, lures based on vertebrate olfactory signals have been developed to attract and trap triatomines (Bouzada et al., 2023; Guidobaldi & Guerenstein, 2013). Vertebrate breath, skin, sweat, urine and excreta contain a wide variety of volatile molecules (Guerenstein & Guérin, 2001; Guerenstein & Lazzari, 2009; Otálora‐Luna & Guérin, 2014). Carbon dioxide (CO_2_), released during host respiration, is detected by triatomines, especially at night (Bodin et al., 2008). Similarly, compounds in human sweat, such as lactic acid, isobutyric acid, and 1‐octen‐3‐ol, are known to attract triatomines (Barrozo & Lazzari, 2004; Guerenstein & Guérin, 2001; Lazzari et al., 2013). Nonanal, a compound found in sheep wool and chicken feathers, has been shown to increase locomotor activity in *T. infestans* (Klug) (Guerenstein & Guérin, 2001). Auffray et al. (2023) demonstrated that the hematophagous red mite *Dermanyssus gallinae* was attracted to a mixture of R‐1‐octen‐3‐ol, octanal, nonanal, (E)‐2‐nonenal and nonanoic acid, whilst these compounds were not attractive when tested individually. Additionally, a 40:60 mixture of nonanal and hexanal was found to be attractive to *T. infestans* in laboratory bioassays (Rojas de Arias et al., 2023). Previous studies have shown a synergistic effect of mixtures containing nonanal and/or octenol on triatomines (Alavez‐Rosas et al., 2024) or other insects (Cao et al., 2024; Galassi & Audino, 2023; Saveer et al., 2023). For instance, in a study by Campuzano‐Granados and Malo (2018), nonanal released by female opossums (*Didelphimorphia* sp.) was found to be attractive to *T. dimidiata* nymphs. Whilst these compounds may be effective individually, their combination significantly increases their effectiveness in attracting triatomines (Guidobaldi & Guerenstein, 2013). Recently, *Rhodnius prolixus* (Ståhl) and *T. infestans* showed attraction when exposed to a mixture of lactic acid, hexanoic acid, and ammonium hydroxide. Additionally, *T. pallidipennis* nymphs infected with *T. cruzi* showed an increased preference for the same odorant mixture compared to non‐infected triatomines (Ramírez‐González et al., 2019). Identifying chemical compounds that attract triatomines could enable the development of lures for detection and control devices (Bouzada et al., 2023). The objective of this study was to identify a mixture that could serve as an effective bait for monitoring and mass trapping projects aimed at controlling populations of *T. pallidipennis*, a significant vector of *T. cruzi* in Mexico.

## MATERIALS AND METHODS

### T. pallidipennis *nymphs*

*T. pallidipennis* specimens were originally collected in Oaxtepec, Morelos, Mexico, and have been maintained since 1998 at the Laboratorio de Biología de Parásitos, Departamento de Microbiología y Parasitología, Facultad de Medicina, UNAM (DMP‐FM‐UNAM) under controlled conditions of 28°C, 60% RH and a 12:12 L/D photoperiod. The colony is renewed every 6 months by adding new adult individuals (male and female).

### Nymph infection

To infect *T. pallidipennis* specimens with *T. cruzi*, the insects were allowed to feed on CD‐1 mice previously inoculated with 40 μL (20,000 parasites/mL) of the *T. cruzi* strain ITRI/MX/12/MOR (Morelos), which belongs to the TcI genetic group (Cordero‐Montoya et al., 2019). This strain was first isolated from a male *T. pallidipennis* specimen collected in the state of Morelos, Mexico, in 2012. The strain has been maintained by successive passages in female CD‐1 mice at the Laboratorio de Biología de Parásitos (DMP‐FM‐UNAM), as described by Favila‐Ruiz et al. (2018). Triatomines were allowed to feed on infected mice at 20 days post‐infection, which corresponds to the early exponential phase of *T. cruzi* growth. Based on parasitemia levels in mice, each insect ingested approximately 8000 parasites. Infection in triatomines was ensured by abdominal compression and verified by microscopic examination of tissues at 40× magnification at 15 days post‐infection. Non‐infected insects were allowed to feed on *T. cruzi*–free mice. Triatomines were allowed to feed in groups of three insects per mouse for 20 min at night (Favila‐Ruiz et al., 2018). Third (N3), fourth (N4) and fifth (N5) instar nymphs were used for all assays.

### Chemical compounds

Octenol (1‐octen‐3‐ol, 98% purity, Sigma‐Aldrich, Toluca, Mexico), nonanal (95% purity, Sigma‐Aldrich) and an odorant mixture consisting of ammonium hydroxide (99%, Fermont, Monterrey, Mexico), L‐(+)‐lactic acid (98% purity, Sigma‐Aldrich) and hexanoic acid (99% purity, Sigma‐Aldrich) (in a ratio of 1:0.2:0.4 respectively) (Guidobaldi & Guerenstein, 2013; Ramírez‐González et al., 2019) were used. Hexane (>99% purity, Fermont, Monterrey, Mexico) and distilled water were used as solvents.

### Attraction tests

**FIGURE 1:**
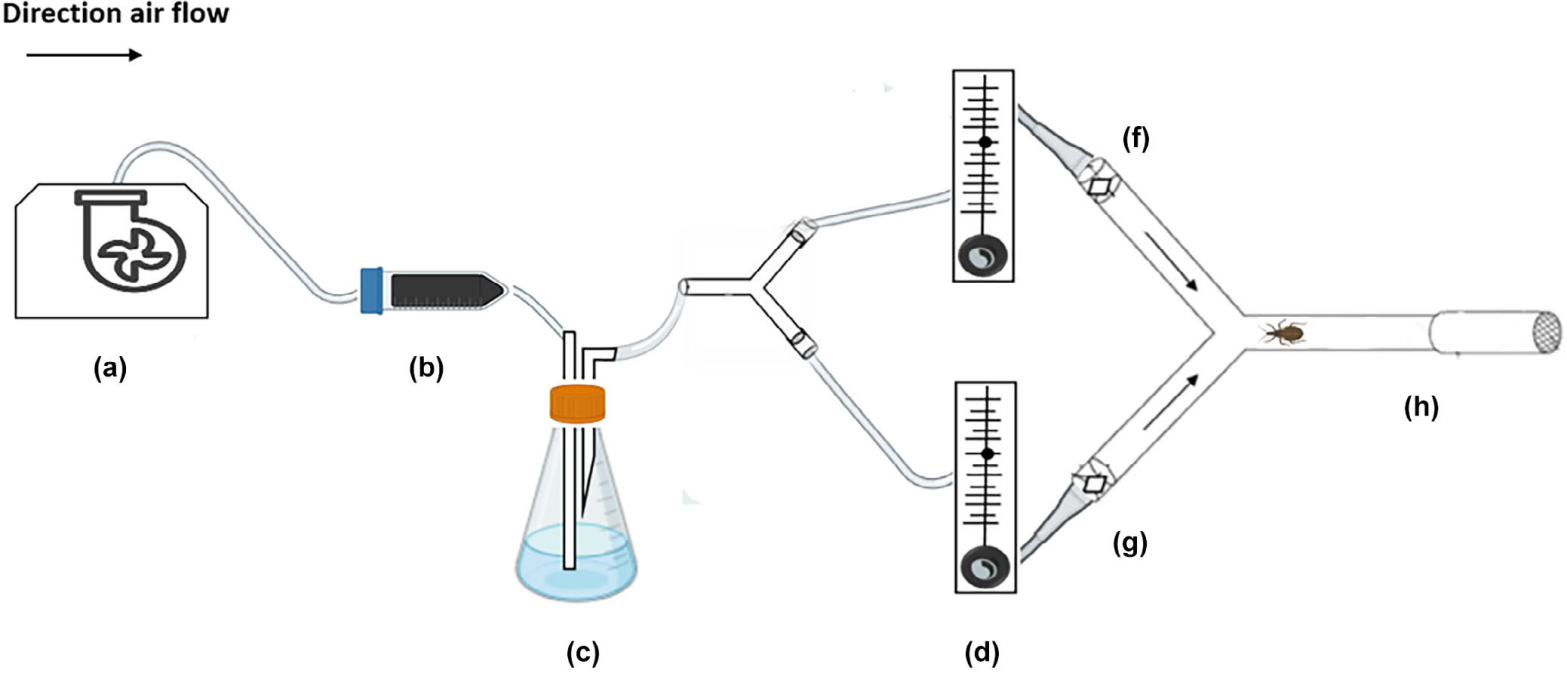
Schematic of a Y‐shaped glass tube olfactometer. The arrows indicate the direction of air flow, which was generated by an air pump (a) connected to an activated charcoal filter (b) and a humidifier (c) and regulated by a flow meter (d). The test compounds were placed on a square of filter paper (g and f) at the top of each arm (odorant chamber), which was connected to flowmeter. A single insect was placed at the base of the olfactometer where it was free to move to the Y‐shaped intersection and then select an arm to enter (h).

The attractive response in triatomines (either non‐infected or infected with *T. cruzi*) to volatile compounds was evaluated in a Y‐type olfactometer, consisting of a Y‐shaped glass tube with an internal diameter of 2.5 cm; the base and each arm of the olfactometer were 12‐cm long and set at an angle of 45°. The olfactometer was placed horizontally, and a stream of humidified, charcoal‐purified air, generated by a vacuum pump (L‐7920‐10, Cole‐Parmer, Vernon Hills, IL, USA) and regulated by a flowmeter at a rate of 0.5 L/min, was passed through each lateral arm (Figure 1). To evaluate the olfactory responses of triatomines to the compounds (nonanal, octenol, and the odorant mixture described above), a piece of filter paper (1 × 1 cm, Maidstone, UK) was placed in each arm of the tube 1 min before the experiment. The selected dose of the compound or mixture to be evaluated was placed on the paper to allow the solvent to evaporate. In a first assay, nonanal and octenol were evaluated individually at doses of 1, 10 and 100 ng, using hexane as solvent (Alavez‐Rosas et al., 2024; Barrozo & Lazzari, 2004; Saveer et al., 2023), in both *T. cruzi*–infected and non‐infected nymphs. Pure hexane was included as a negative control. In a second assay, the odorant mixture of lactic acid, hexanoic acid and ammonium hydroxide was evaluated at doses of 1, 10 and 100 ng (summing all constituents), using distilled water as solvent and as control (Ramírez‐González et al., 2019), in both *T. cruzi*–infected and non‐infected nymphs. In a third assay, 1 ng of the odorant mixture was tested against 1 ng of nonanal or octenol, and 1 ng of octenol was tested against 1 ng of nonanal. Finally, 1 ng of the odorant mixture + 1 ng of nonanal was evaluated against 1 ng of the mixture and against 1 ng of nonanal alone. For all assays, 1 μL of each solution was applied to a square of filter paper and the solvent was allowed to evaporate, 30 s for hexane and 1 min for distilled water.

In each experiment, a single N3, N4 or N5 nymph was placed at the base of the “Y” olfactometer (Figure 1), and its behaviour was recorded for 10 min after exposure to the compounds being tested. The insects were deprived of food for 15 days prior to the bioassays to promote activity.

A choice was regarded as positive if the triatomine reached the end of the olfactometer arm and remained there for at least 45 s. Any specimen that failed to respond within 10 min was excluded from the analysis. Each insect was used only once and then discarded. In each replicate, treatments were alternated on the olfactometer arms to prevent directional or positional bias. Each treatment was repeated on 40 insects. All bioassays were conducted between 6:00 PM and 11:00 PM in a dark room at a temperature of 25 ± 2°C and illuminated by a red light. After each series of observations, the olfactometer was thoroughly washed with detergent, distilled water and 70% ethanol and sterilised at 120°C for 20 min to keep it free of any chemical residues and/or microorganisms.

In the first two experiments, N3, N4 and N5 *T. pallidipennis* nymphs, either *T. cruzi*–infected or non‐infected, were evaluated. In the third and fourth experiments, only N3 and N4 nymphs, either *T. cruzi*–infected or non‐infected, were evaluated, as these stages showed a greater response to all compounds and doses tested.

### Statistical analysis

Data were analysed using the language R v.4.3.1 (Core Team 2023). Data from double‐choice bioassays were compared with the G‐test (*χ* ^2^ with Yates correction), using the DescTools package (Signorell et al., 2016).

## RESULTS

**FIGURE 2:**
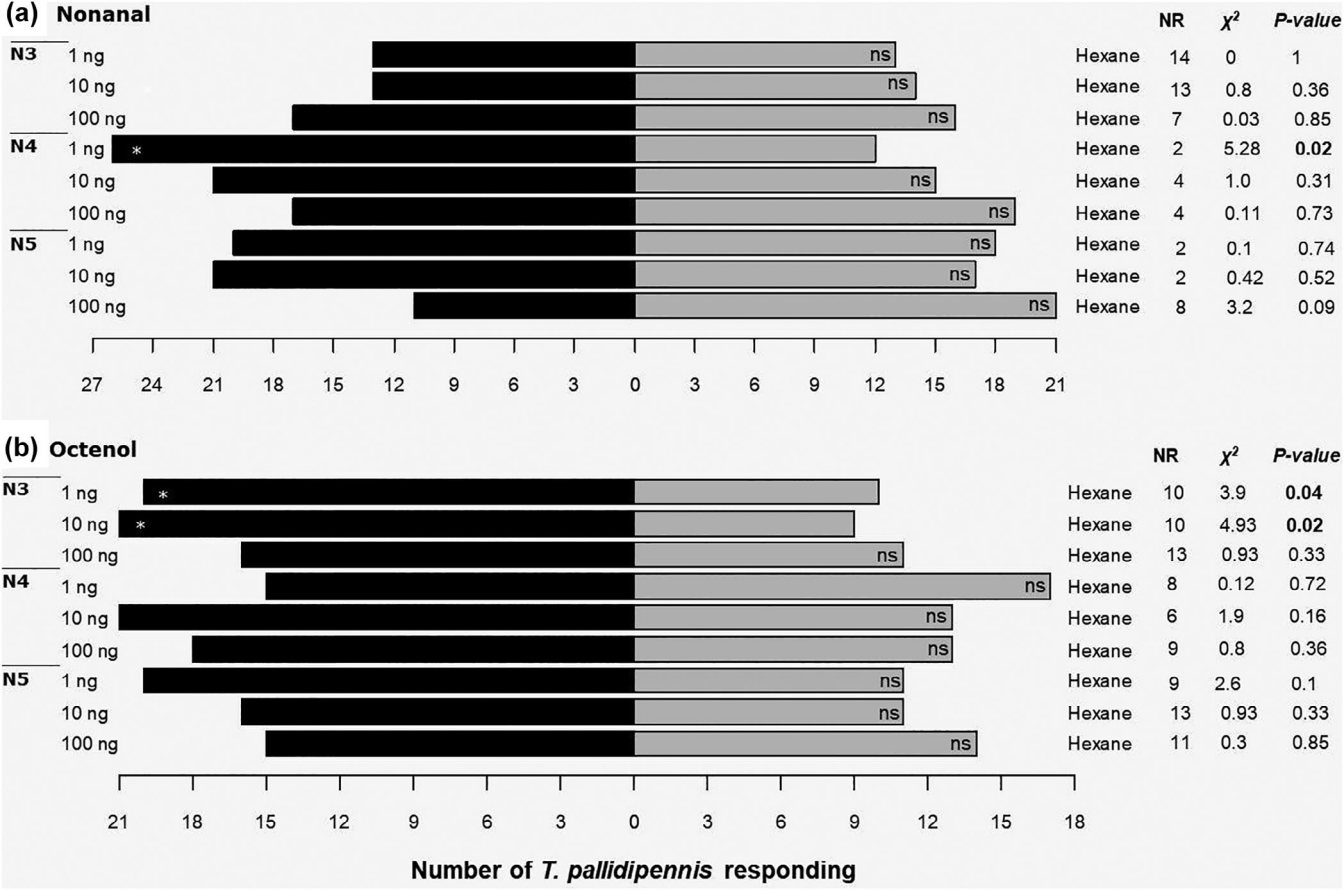
Response of N3, N4 and N5 *T. pallidipennis* nymphs, not infected with *T. cruzi* (*n* = 40) in a Y‐tube olfactometer. Of (a) nonanal or (b) octenol (1‐octen‐3‐ol), 1, 10 and 100 ng were used. Pure hexane was included as a control. NR, no response, indicates the number of specimens that did not respond within 10 min. A *χ* ^2^ test was used, df = 1; ns, not significant; asterisk, significant.

Non‐infected (non‐*T. cruzi*) N3 *T. pallidipennis* nymphs were attracted only to 1 and 10 ng octenol, but not to hexane (control) (Figure 2b). N4 specimens showed a preference for 1 ng nonanal only (Figure 2a). N5 triatomines showed no preference for either compound (nonanal and octenol) at any concentration tested (Figure 2a,b).

**FIGURE 3:**
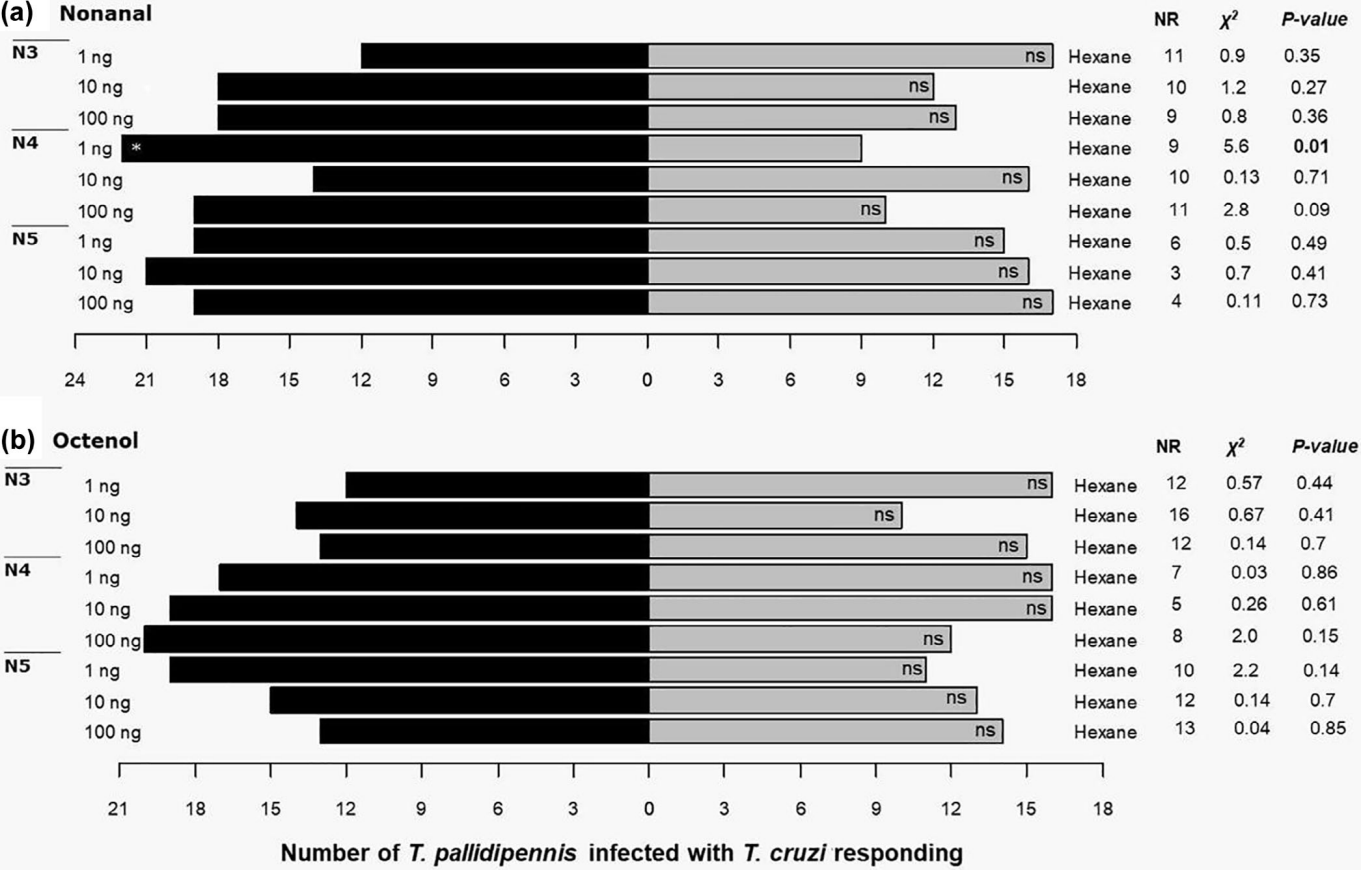
Response of N3, N4 and N5 *T. pallidipennis* nymphs infected with *T. cruzi* (*n* = 40) in a Y‐tube olfactometer. Of (a) nonanal or (b) octenol (1‐octen‐3‐ol), 1, 10 and 100 ng were used. Pure hexane was included as a control. NR, no response, indicates the number of specimens that did not respond within 10 min. A *χ* ^2^ test was used, df = 1; ns, not significant; asterisk, significant.

*T. cruzi*–infected, N3 *T. pallidipennis* specimens showed no preference for any of the compounds evaluated at different doses (Figure 3a,b). N4 nymphs were more attracted to 1 ng of nonanal than to the control (Figure 3a). N5‐infected triatomines showed no preference for either compound (nonanal and octenol) at the concentrations evaluated (Figure 3a,b).

**FIGURE 4:**
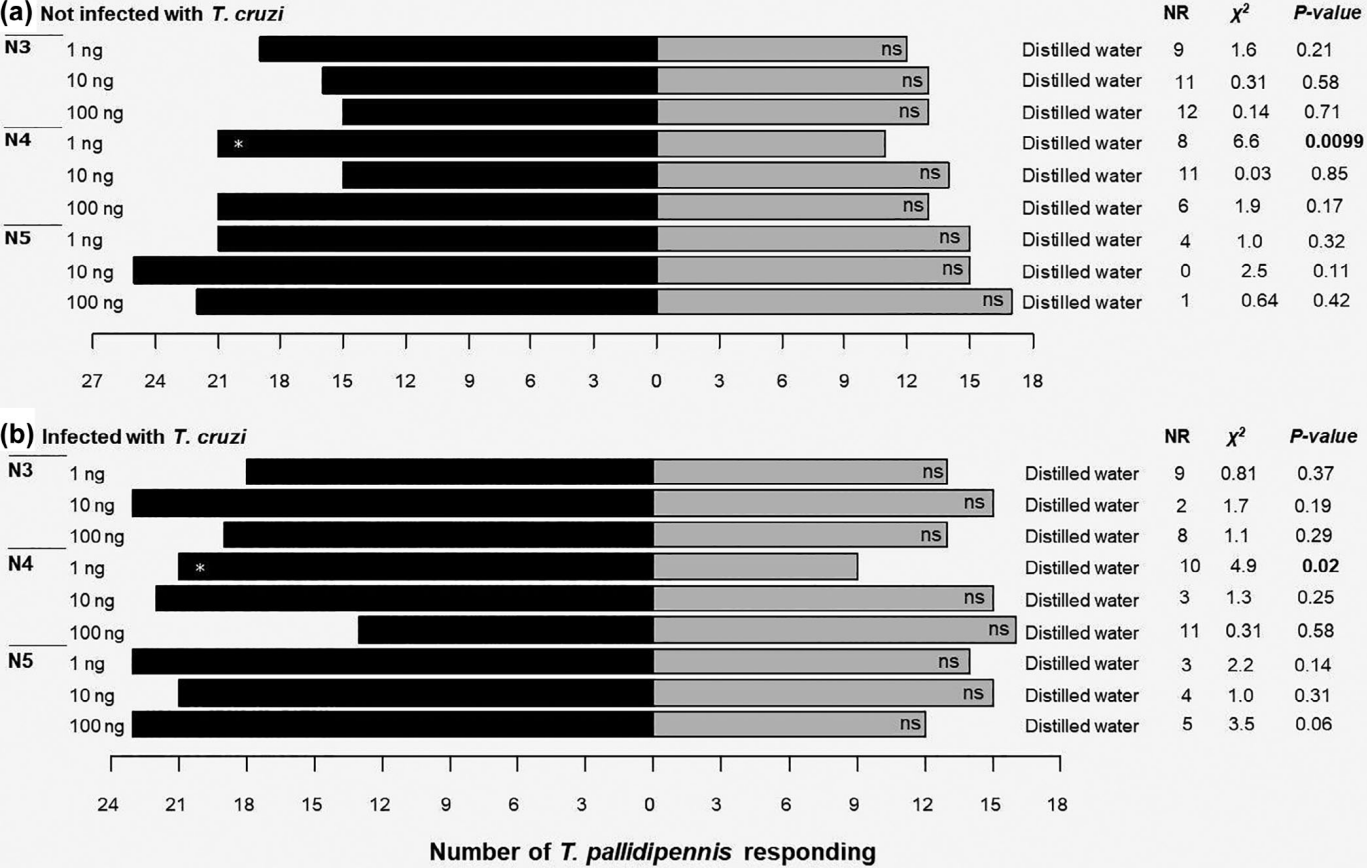
Response of N3, N4 and N5 *T. pallidipennis* nymphs, either non‐infected or infected with *T. cruzi* (*n* = 40) in a Y‐tube olfactometer. Response of (a) non‐infected nymphs and of (b) infected nymphs to an odorant mixture of lactic acid, ammonium hydroxide and hexanoic acid in amounts of 1, 10 and 100 ng. Distilled water was included as a control. NR, no response, indicates the number of specimens that did not respond within 10 min. A *χ* ^2^ test was used, df = 1; ns, not significant; asterisk, significant.

Non‐infected and *T. cruzi*–infected N3 nymphs showed no preference for odorant mixture (ammonium hydroxide + lactic acid + hexanoic acid, in a ratio 1:0.2:0.4 respectively) at any dose (1, 10 and 100 ng) (Figure 4a,b). Non‐infected and *T. cruzi*–infected N4 nymphs were more attracted to 1 ng of the odorant mixture than to distilled water (control) (Figure 4a,b). Both non‐infected and infected N5 nymphs showed no preference for the odorant mixture at any dose (Figure 4a,b).

**FIGURE 5:**
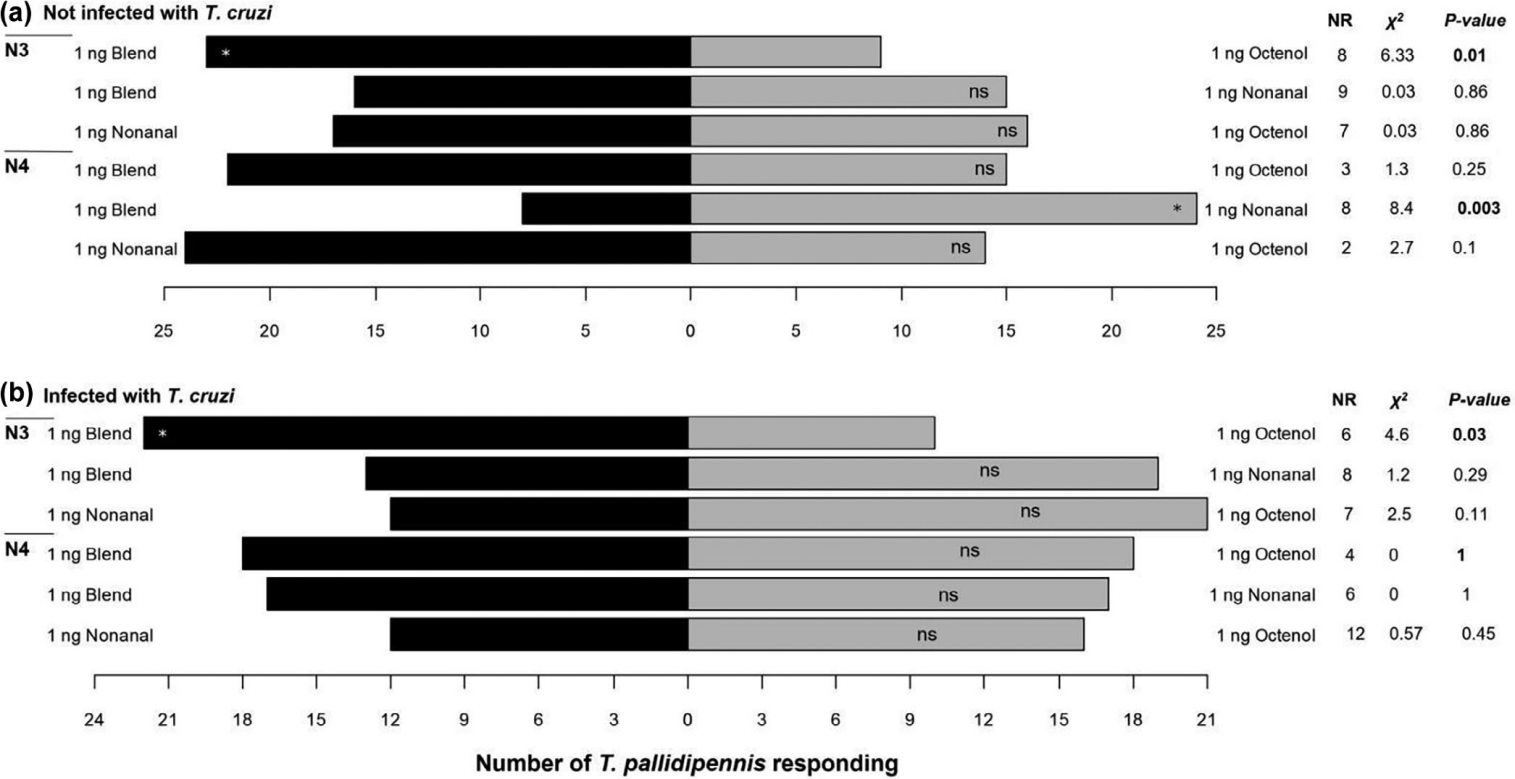
Response of N3 and N4 *T. pallidipennis* nymphs, either non‐infected or infected with *T. cruzi* (*n* = 40) in a Y‐tube olfactometer. Response of (a) non‐infected nymphs and of (b) infected nymphs to an odorant mixture of lactic acid, ammonium hydroxide, and hexanoic acid (1 ng) against nonanal or octenol (1 ng), and nonanal against octenol (1 ng). NR, no response, indicates the number of specimens that did not respond within 10 min. A *χ* ^2^ test was used, df = 1; ns, not significant; asterisk, significant.

Based on these preliminary results, nonanal, octenol and the odorant mixture were evaluated at a dose that proved to be attractive (1 ng) to N3 and N4 nymphs, the stages that showed the greatest olfactory response (Figures 2, 3, 4). Non‐infected and *T. cruzi*–infected *T. pallidipennis* N3 nymphs showed a greater preference for the odorant mixture than for octenol (Figure 5a,b). Non‐infected N4 nymphs were more attracted to nonanal than to the odorant mixture (Figure 5a). Infected N4 nymphs showed no preference for either compound or the odorant mixture (Figure 5b).

**FIGURE 6:**
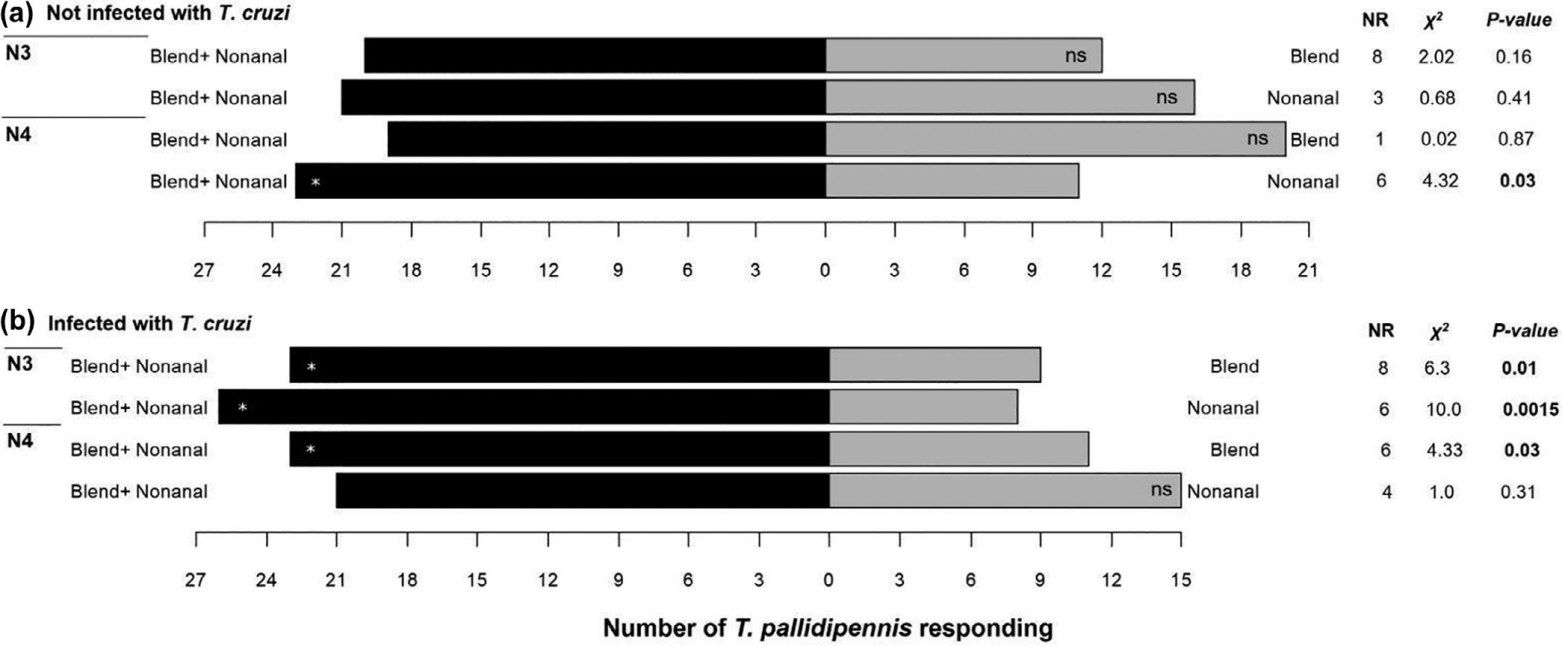
Response of N3 and N4 *T. pallidipennis* nymphs, either non‐infected or infected with *T. cruzi* (*n* = 40) in a Y‐tube olfactometer. Response of (a) non‐infected nymphs and of (b) *T. cruzi*–infected nymphs to an odorant mixture of lactic acid, ammonium hydroxide and hexanoic acid + nonanal (1 ng each) versus either the odorant mixture (1 ng) or nonanal (1 ng) alone. NR, no response, indicates the number of specimens that did not respond within 10 min. A *χ* ^2^ test was used, df = 1; ns, not significant; asterisk, significant.

A combination of 1 ng of the odorant mixture plus 1 ng of nonanal was then evaluated against the odorant mixture and nonanal alone. Acting synergistically, the combination odorant mixture + nonanal was more attractive to *T. cruzi*–infected N3 nymphs than either the odorant mixture or nonanal alone (Figure 6b). However, non‐infected N3 nymphs showed no preference for either compound (Figure 6a). Non‐infected N4 nymphs were more attracted to the mixture + nonanal than to nonanal alone (Figure 6a). In contrast, *T. cruzi*–infected N4 nymphs were more attracted to the mixture + nonanal than to the mixture alone (Figure 6b).

## DISCUSSION

Our results indicate that the response of triatomines to chemical signals varies depending on the concentration of the compound and the developmental stage of the insects. Nonanal, octenol and an odorant mixture that consisted of ammonium hydroxide, lactic acid and hexanoic acid (in a ratio 1:0.2:0.4, respectively) were more attractive at low doses (1 ng) to both *T. cruzi*–infected and non‐infected insects. This pattern of behaviour also appeared to be influenced by developmental stage, as N3 and N4 nymphs showed a greater attraction to these compounds than N5 nymphs.

Several compounds with potential value as attractants have been identified in the volatiles of vertebrate hosts and in the sex and aggregation pheromones of triatomines (Manrique et al., 2023). These compounds are known for their ability to attract hemipterans and other hematophagous insects. For instance, nonanal was detected among more than 10 volatile compounds in the faeces of *T. cruzi*–infected and non‐infected *T. pallidipennis* specimens and was found to be attractive to this vector (Alavez‐Rosas et al., 2024). Nonanal has also been identified as a major component in the faeces of *T. dimidiata* (Galvez‐Marroquin et al., 2018) and bedbugs (*Cimex lectularius*, Siljander et al., 2008). The presence of nonanal has been reported in the volatile compounds emitted by *T. infestans* during copulation (Fontan et al., 2002). In this context, sticky traps with nonanal have proven sensitive in detecting the presence of *T. infestans* in houses compared to manual searches (Rojas de Arias et al., 2012).

Another important compound is octenol, which is also released by vertebrate hosts and has found to be attractive to various hematophagous arthropods, including mosquitoes (Dormont et al., 2021), bedbugs (Akhoundi et al., 2023), tsetse flies (Gikonyo et al., 2002) and lice (Galassi & Audino, 2023), in some cases in synergy with nonanal. These odorants, even when acting individually, can induce activation and/or attraction in triatomines. Guerenstein and Guérin (2001) observed that nonanal increased locomotor activity in *T. infestans*. Similarly, octenol was found to elicit an attractive response in *T. infestans* nymphs when tested individually (Guerenstein & Guérin, 2001).

In our study, *T. pallidipennis* nymphs were attracted to octenol and nonanal, but only at low doses of both compounds. These results are consistent with those reported by Galassi et al. (2018), where the response of lice to nonanal was dependent on the concentration, being repellent at high doses (1 and 10 μg) and attractive at low doses (0.01 and 0.1 μg). In the case of triatomines, nonanal has been shown to attract male *T. infestans* at low doses (0.01–0.1 μg) (Fontan et al., 2002). Nonanal was also found to attract *T. infestans* nymphs at doses of 1 and 5 μg, whilst adult males and females were more attracted to a dose of 1 μg (Rojas de Arias et al., 2023). Thus, nonanal could signal the presence of a host to the insects, in addition to influencing the sexual and aggregation behaviour of triatomines. On the other hand, when Barrozo and Lazzari (2004) tested five doses (1, 10, 100, 1000 and 10,000 μg) of octenol on *T. infestans*, the attraction was observed only at an intermediate dose (100 μg).

Interestingly, insects usually respond better to mixtures of specific compounds emitted by the host than to single compounds (Barrozo & Lazzari, 2004; Manrique et al., 2023). For example, Guidobaldi and Guerenstein (2013) observed that a bait containing a mixture of ammonia, L‐lactic acid and hexanoic acid significantly increased the capture of N3 nymphs of both *R. prolixus* and *T. infestans*. However, no attraction was observed when the mixture components were evaluated individually or in binary combinations, suggesting a synergistic effect of the three compounds. This is consistent with our results: When the odorant mixture (ammonium hydroxide, lactic acid, and hexanoic acid) was tested against nonanal and octenol, the odorant mixture ranked first in triatomine preference, followed by nonanal. Octenol was not as attractive as the mixture or nonanal. These findings led us to select nonanal as the prime candidate for testing its potential to enhance the mixture's attractiveness. Our results showed that the mixture and nonanal acted synergistically to attract triatomines, with this effect being more pronounced in *T. cruzi*–infected specimens. A recent study also found that a 40:60 mixture of nonanal and hexanal was more effective in attracting *T. infestans* than nonanal alone (Rojas de Arias et al., 2023). Similarly, in a lepidopteran, it was found that nonanal alone does not attract male or female *Spodoptera frugiperda* (J.E. Smith) (Lepidoptera: Noctuidade) in the field, but in synergy with the sex pheromone of this species, it proved to be more attractive than the sex pheromone alone (Saveer et al., 2023). On the other hand, the attractant effect of octenol in our study is unclear, warranting further experiments to evaluate its potential as an attractant. In future studies, baits for trapping *T. pallidipennis* specimens based on a combination of the evaluated compounds (odorant mixture + nonanal, odorant mixture + octenol, odorant mixture + nonanal + octenol, nonanal + octenol) will be assessed to study possible synergistic and antagonistic effects among them. Whilst our results are promising, the total emission rates and proportions of compounds in our experiments may differ from those obtained using a different dispenser type, making direct comparisons challenging. The type of dispenser (septum, membrane and solid matrix dispensers) is crucial for maximising the efficacy of volatile compounds as insect baits, as it influences both release rates and insect responses (Klassen et al., 2023; Nielsen et al., 2019). The efficacy of the tested mixture should be evaluated in field or semi‐field trials using other dispenser types. Optimised baits could facilitate the development of “attraction and infection” systems based on biological control agents such as the entomopathogenic fungus *Metarhizium anisopliae* (Toriello et al., 2022).

The effects of *T. cruzi* infection on triatomine biology are well documented. This pathogen is known to alter the phenotype, behaviour and physiology of triatomines to its own advantage, thereby increasing its transmission capacity to the final host (Poulin & Maure, 2015). For example, May‐Concha et al. (2022) found that *T. cruzi* infection altered the antennal phenotype in natural populations of *T. dimidiata*. Other studies have shown that *T. cruzi*–infected triatomines detect their vertebrate hosts more quickly, exhibit increased locomotor capacity, defecate sooner, deposit larger amounts of faeces and bite more frequently compared to non‐infected insects (Botto‐Mahan et al., 2006; Marliére et al., 2015; Pereyra et al., 2020; Ramírez‐González et al., 2019). Most of these responses could be related to the parasite burden and the nutritional status of the insects, with a higher burden leading to greater vector efficiency (Chacón et al., 2022; Estay‐Olea et al., 2020; Marliére et al., 2021). However, other studies have reported that *T. cruzi* infection in *R. prolixus* and *T. infestans* does not result in differences in biting attempts (Pereyra et al., 2020; Takano‐Lee & Edman, 2002). Additionally, *T. cruzi* infection has been reported to decrease the locomotor activity of *R. prolixus* specimens (Marliére et al., 2015). Thus, the great variability in the effects of *T. cruzi* on vectors depends on the triatomine species, parasite load, the discrete taxonomic unit (DTU) or genotype of *T. cruzi* isolates (Flores‐Villegas et al., 2022), the age and sex of the insect and other physiological and environmental factors (Loshouarn & Guarneri, 2024). In a previous study, Ramírez‐González et al. (2019) found that younger (N3) *T. pallidipennis* specimens infected with a Mexican *T. cruzi* strain (Morelos) showed a greater attraction to the odorant mixture (ammonium hydroxide, lactic acid and hexanoic acid) compared to non‐infected insects.

In contrast to those findings, our study revealed that both non‐infected and *T. cruzi*–infected N3 nymphs showed no preference for the odorant mixture at any dose. However, N4 nymphs were more attracted to 1 ng of the odorant mixture compared to the control, whilst N5 nymphs showed no preference for the odorant mixture at any dose. It should be noted that the olfactometry system used in the Ramírez‐González et al. (2019) study included a dual‐choice device with a drop trap, designed to account for the behaviour of *T. infestans*, which tend to drop from the ceiling when detecting attractant odours from below (Guidobaldi & Guerenstein, 2013; Guidobaldi & Guerenstein, 2016; Rojas de Arias et al., 2023). Given that *T. pallidipennis* has a predominantly peridomiciliary distribution likely due to the presence of shelters in stone fences that allow free access to farm animals and pets (Alejandre‐Aguilar et al., 2023), we opted to use a Y‐type olfactometer. Additionally, the possible synergistic effect of the odorant mixture and nonanal at different concentrations was not investigated by Ramírez‐González et al. (2019). Additionally, the Y‐tube has been widely used as a dual‐choice bioassay, which was necessary in this study, as it was essential to evaluate the synergy of mixtures and compounds against mixtures or compounds alone, something that cannot be achieved with a linear tube (Alavez‐Rosas et al., 2024; Mérida‐Torres et al., 2023).

The olfactory differences observed in the N3, N4, and N5 nymphal stages may result from ‘olfactory plasticity’, which refers to the ability of insects to adjust their responses to odours based on their physiological state. Insects can modify their olfactory response according to developmental stage, feeding status and circadian rhythms as a strategy to optimising resource seeking for survival and reproduction (Gadenne et al., 2016; Reisenman et al., 2013). For instance, in the case of the bell pepper weevil (*Anthonomus eugenii*), specificity towards host plant odours increases as the insects mature, suggesting that older insects are more selective in their foraging (Addesso & McAuslane, 2009). Our results suggested that less mature developmental stages are more susceptible to the effects of the parasite (Poulin et al., 1994). Host may exhibit resistance to parasite manipulation, especially when infections impose burdens on survival and fecundity (Daoust et al., 2015). It is likely that N5 nymphs only risk exposure and foraging if they sense the presence of chemical cues indicating a suitable host. Additionally, resistance to parasite manipulation by vectors could help prevent predation risks and the loss of nutritional resources essential for their own growth and survival (Estay‐Olea et al., 2020).

## CONCLUSION

Our study suggests that infection with *Trypanosoma cruzi* (Morelos isolate) may enhance the olfactory perception of *T. pallidipennis* to a mixture of compounds but not to single compounds. This heightened perception could enable infected triatomines to more effectively locate food sources, whether human or domestic/farm animals, thereby potentially facilitating the vector transmission of *T. cruzi*. We identified an odorant mixture (ammonium hydroxide, lactic acid and hexanoic acid) that acts synergistically with nonanal to induce an attractant effect, which could be valuable in designing baits for monitoring or control devices. This finding could contribute to the development of effective ‘attract and infect’ systems based on biocontrol agents.

## AUTHOR CONTRIBUTIONS

**Berenice Jiménez‐Santiago:** Conceptualization; methodology; investigation; visualization; writing – review and editing; writing – original draft. **Any Laura Flores‐Villegas:** Writing – review and editing; writing – original draft; methodology; investigation. **Samuel Cruz‐Esteban:** Methodology; writing – original draft; writing – review and editing; formal analysis. **Margarita Cabrera‐Bravo:** Writing – review and editing; writing – original draft; investigation. **Conchita Toriello:** Writing – original draft; writing – review and editing; funding acquisition.

## CONFLICT OF INTEREST STATEMENT

The authors declare no conflicts of interest.

## ETHICS STATEMENT

This project was approved by the Ethics and Research Committee of the Faculty of Medicine, National Autonomous University of Mexico, permit FM/DI/080/2023.

## Data Availability

Raw sequence read files are deposited at https://doi.org/10.5281/zenodo.13864637.
